# Reliable Detection of Herpes Simplex Virus Sequence Variation by High-Throughput Resequencing

**DOI:** 10.3390/v9080226

**Published:** 2017-08-16

**Authors:** Alison M. Morse, Kaitlyn R. Calabro, Justin M. Fear, David C. Bloom, Lauren M. McIntyre

**Affiliations:** University of Florida Genetics Institute, Department of Molecular Genetics & Microbiology, University of Florida College of Medicine, Gainesville, FL 32611, USA; ammorse@ufl.edu (A.M.M.); k.calabro@ufl.edu (K.R.C.); Justin.m.fear@gmail.com (J.M.F.); dbloom@ufl.edu (D.C.B.)

**Keywords:** de novo assembly, reference-based assembly, high-throughput sequencing, single-nucleotide polymorphisms

## Abstract

High-throughput sequencing (HTS) has resulted in data for a number of herpes simplex virus (HSV) laboratory strains and clinical isolates. The knowledge of these sequences has been critical for investigating viral pathogenicity. However, the assembly of complete herpesviral genomes, including HSV, is complicated due to the existence of large repeat regions and arrays of smaller reiterated sequences that are commonly found in these genomes. In addition, the inherent genetic variation in populations of isolates for viruses and other microorganisms presents an additional challenge to many existing HTS sequence assembly pipelines. Here, we evaluate two approaches for the identification of genetic variants in HSV1 strains using Illumina short read sequencing data. The first, a reference-based approach, identifies variants from reads aligned to a reference sequence and the second, a de novo assembly approach, identifies variants from reads aligned to de novo assembled consensus sequences. Of critical importance for both approaches is the reduction in the number of low complexity regions through the construction of a non-redundant reference genome. We compared variants identified in the two methods. Our results indicate that approximately 85% of variants are identified regardless of the approach. The reference-based approach to variant discovery captures an additional 15% representing variants divergent from the HSV1 reference possibly due to viral passage. Reference-based approaches are significantly less labor-intensive and identify variants across the genome where de novo assembly-based approaches are limited to regions where contigs have been successfully assembled. In addition, regions of poor quality assembly can lead to false variant identification in de novo consensus sequences. For viruses with a well-assembled reference genome, a reference-based approach is recommended.

## 1. Introduction

With the advent of high-throughput sequencing (HTS), genome-wide sequencing of DNA viruses such as the herpesviruses has been much more affordable. This has resulted in the generation of complete genome sequences of a number of herpes simplex virus (HSV) laboratory strains, as well as more primary human clinical isolates [1,2,3]. In addition, HTS has opened the door to analyses of recombinants for secondary mutations [4], and analyses of intra-strain variation have become an increasingly important tool in understanding viral biology [5]. While HTS technology is becoming mature and more accessible, bioinformatic strategies for the assembly of complete genomes are made more complicated due to the existence of repeats and arrays of reiterated sequences that are present in many viral genomes [6]. In addition, there is inherently greater genetic variation in isolates of viruses and other microorganisms, which presents an additional challenge to many existing sequence assembly pipelines [7]. There are two primary protocols used to assemble genomes and to detect variations: one takes a reference-based approach and the other a de novo assembly approach.

Reference-based variant discovery is restricted to species with a well-curated reference genome or to species closely-related to a species with a well-curated reference genome [8]. The more closely related the reference genome is to the sequenced genome, the more likely it is for reads to be correctly aligned [8]. As sequence identity decreases in regions of increased genetic diversity, it is more likely that reads will either not align or, more critically, align to incorrect locations [8]. The use of longer paired-end reads can reduce this issue, but alignment and variant calling in repetitive regions remains challenging. In addition to real polymorphisms, alignment algorithms must also accommodate artifacts due to bias in hexamer mispriming, GC content, polymerase chain reaction (PCR), and sequencing that cause differences between reads and the reference genome [9,10,11]. Alignment algorithms also need to generate an accurate mapping quality score for each read since these are used in variant determination with their associated probabilities [12].

Concern with reference-based approaches has led to the development and use of de novo assembly algorithms (reviewed in [13,14]). The assembled consensus, from here on ‘contig’ sequences, is the genetic average of all variants within the population, including any larger structural changes that may be present [15]. For more diverse regions in a sequenced genome relative to a reference, the de novo assembly of an individual strain can result in a higher number of valid read re-alignments, at the potential expense of variant detection relative to a curated reference [16]. However, there are important costs and considerations associated with de novo assembly and variant calling. A high-quality assembly requires a sufficient number of error-free reads across the genome to minimize assembly artifacts; genomic repeat regions can be difficult to resolve and may get compacted or collapsed into single regions, and contamination can be an issue, particularly with shorter read lengths [13]. Finally, it is unlikely that the entire genome (even one the size of a virus) will be assembled completely, and so, some comparison to a reference strain to order contigs and fill in gaps will be needed. Altogether, the generation of a high-quality assembly involves several manual curation steps and may therefore be a protracted and time-consuming process.

**Figure 1:**
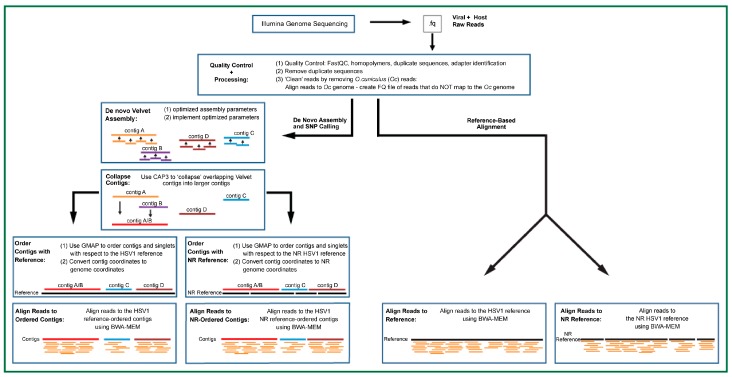
Strategies for individual viral sequencing: a comparison of reference-based versus de novo assembly and alignments for downstream variant calling. FASTQ files contain read sequences for both herpes simplex virus (HSV) 1 and the rabbit (*Oryctolagus cuniculus*, (*Oc*)) skin cell line used to propagate the virus. Read quality was evaluated, and reads were aligned to the *O. cuniculus* reference to remove the host reads. (Left side) Reads were assembled de novo using Velvet to create contigs and CAP3 to collapse overlapping Velvet contigs into longer contigs. In preparation for variant calling, contigs were aligned to the full-length HSV1 reference (GenBank Accession Number: NC_001806) and the HSV1 non-redundant (NR)-reference (Supplementary File 1). The set of putative HSV1 reads used to create the contigs was aligned to the contigs. (Right side) Reads were aligned to the full-length HSV1 (GenBank Accession Number: NC_001806) reference and the HSV1 NR-reference (Supplementary File 1). BWA-MEM: Burrows-Wheeler Aligner; GMAP: Genomic mapping and alignment program.

Here, we present a comparative evaluation of each of these protocols for use in assembling HTS-sequenced HSV1 (Figure 1). After read quality control and removal of host read contamination, reads are assembled into consensus sequences, or ‘contigs’, using a de novo assembly algorithm [17]. Reads are then mapped against the contigs for variant identification within the de novo assembly. Variants identified by de novo assembly were compared to variants identified by mapping reads directly against the HSV1 reference. Mapping against the HSV1 reference was carried out in two ways: reads were mapped against the HSV1 reference genome or against a non-redundant (NR) version of the reference where sequence records for the internal repeat long (IRL) and terminal repeat short (TRS) blocks from the HSV1 reference were removed and variants identified. The use of an NR-reference has been successfully implemented in the genetic diversity study of human cytomegalovirus [18]. The variants identified from the different approaches were concordant 85% of the time. An additional 15% of the variants are identified in the reference-based approach in regions where there are gaps in the contig assembly. 

## 2. Materials and Methods 

### 2.1. Viruses

HSV1 strain 17syn+ was obtained from Jack G. Stevens (University of California, Los Angeles (UCLA), Los Angeles, CA, USA). HSV∆CTRL2-10.1 (for simplicity, referred to here as 17∆CTRL2) was constructed by homologous recombination by co-transfection of unit length HSV1 strain 17syn+ DNA with a recombination plasmid containing a 135 bp deletion of the CTRL2 CTCF motif cluster [19]. Both viruses were propagated on rabbit skin cells (RCS)—obtained from Bernard Roizman (Marjorie B. Kovler Viral Oncology Laboratories, University of Chicago, Chicago, IL, USA)—in Modified Eagle’s Medium (Life Technologies, Carlsbad, CA, USA) supplemented with 5% calf serum, 250 U penicillin, 250 µg/mL streptomycin, 2.5 µg/mL amphotericin B and 292 µg/mL l-glutamine/mL. We selected the 17∆CTRL2 virus as a control to ensure that the deletion in a repetitive region could be detected.

### 2.2. DNA Extraction, Illumina Library Preparation and Sequencing

Unit length genomic viral DNA was prepared from HSV1 17syn+ and 17∆CTRL2 from infected rabbit skin cells (RSCs) as previously described [20]. In summary, the infected cells were pelleted and resuspended in hypotonic lysis buffer (10 mM Tris-Cl, pH 8.0; 10 mM ethylenediaminetetraacetic acid (EDTA); 0.25% sodium deoxycholic acid (NaDOC); 0.5% tergitol-type NP-40 (NP-40), and the cell nuclei were pelleted at 800× *g* for 10 min and discarded. Sodium dodecyl sulfate (SDS) and proteinase K were added to the cytoplasmic fraction (containing the packaged virions) to final concentrations of 1% and 1 mg/mL, respectively, and incubated for 1 hat 50 °C. The DNA was then extracted by phenol and phenol/chloroform extraction, followed by ethanol precipitation. Barcoded sequencing libraries (allowing for six libraries per flow cell lane) were prepared using the KAPA LTP Library Preparation Kit according to the manufacturer’s directions (Kapa Biosystems, Wilmington, MA, USA). Libraries were sequenced using paired-end, 50 bp sequencing protocols on an Illumina HiSeq2500 (Florida State University, Tallahassee, FL, USA).

### 2.3. Illumina Raw Read QC and Processing

Overall quality of the FASTQ files was verified using FastQC [21]. Using PHRED quality scores as a measure of confidence in base calls by the sequencer [22,23], HSV1 17syn+ and 17∆CTRL2 read qualities were high; the most frequently observed mean sequence quality score was greater than PHRED 27. Host (rabbit) contamination was removed using Bowtie1 (paired end, three mismatches, unique) [24] to identify sequences aligning to the host genome (OryCun2.0, GenBank Accession Number: GCA_000003625.1). Ambiguous and unaligned reads were combined, and identical duplicate reads were removed (fastqSplitDups.py Supplementary File 2), leaving 1 copy of each duplicate read and all of the unique reads in the resulting FASTQ file. We refer to these resulting reads as ‘host-processed’.

### 2.4. Creation of HSV1 Non-Redundant Genome

Using the junctions of the repeat long (RL)-unique long (UL) and repeat short (RS)-unique short (US) segments to mark the boundaries of the repeat regions and unique regions, the HSV1 reference (GenBank Accession Number: NC_001806) was divided into sequence ‘blocks’ such that each block generated an individual FASTA sequence record within the resulting FASTA file. The HSV1 NR-reference was constructed by removing the sequence records for the IRL and TRS blocks (Supplementary File 1).

### 2.5. De Novo Contig Assembly

Host-processed reads were used as input into the de novo assembly algorithm. Since the PHRED quality score at the 50 bp position was greater than the standard minimum PHRED quality score of 20 used for read trimming, no read trimming was performed. VelvetOptimiser 2.1.7 [25] was used to identify the optimal Velvet parameters for de novo assembly of HSV1 from the host-processed distinct reads (hash length 45, expected coverage 278, coverage cutoff 0.2614). Velvet (Version 1.2.10) [17] was run with the optimized parameters. CAP3 (Version 20120705) [26] was used to collapse the Velvet contigs (default settings). The resulting assemblies (Velvet and Velvet + CAP3) were evaluated using Quality Assessment Tool for Genome Assemblies (QUAST) program [27]. Velvet-CAP3 contigs greater than 500 bp were aligned to the HSV1 reference or the HSV1 NR-reference using the BLAST-Like Alignment Tool (BLAT) algorithm [28] followed by maf-convert.py (LAST Version 247) [29] to convert Multiple Alignment Format (MAF) alignment files to Sequence/Alignment Map (SAM) alignment files.

Orienting the mapped contigs greater than 500 bp relative to the HSV1 and HSV1 NR-references was carried out using the Genomic Mapping and Alignment Program (GMAP) alignment tool [30] followed by conversion of the SAM alignment files to sorted Binary SAM format (BAM) files with SAMTools [31]. Contigs (sorted BAM alignment files) were visualized against each reference using Integrated Genomics Viewer (IGV) [32].

### 2.6. Alignment of Host-Processed Reads

Host-processed reads were aligned as paired ends using the BWA-MEM algorithm from the Burrows-Wheeler Aligner software package [33] to (1) the HSV1 reference, (2) the HSV1 NR-reference or (3) the final assembled contigs. A custom Python script (BWASplitSam.py, Supplementary File 3) was created to parse the resulting BWA SAM alignment files into the following sub-files: (1) an SAM alignment file containing paired end reads uniquely mapped in the correct orientation plus reads where only one of the pair is mapped; (2) FASTQ files and a SAM file containing reads mapping ambiguously; (3) a SAM file containing paired end reads mapping in the wrong orientation; (4) a SAM file of reads that map, but are not a primary alignment and (5) FASTQ files of reads that do not map. SAM alignment files containing uniquely-mapped reads were converted into BAM and mpileup files using SAMTools [31].

### 2.7. Variant Discovery

Variant Call Format (VCF, http://samtools.github.io/hts-specs/VCFv4.3.pdf) files were generated using Freebayes (http://arxiv.org/abs/1207.3907v2, v0.9.15) with filtering by VCFfilter (Variant Quality (QUAL) > 20, https://github.com/vcflib/vcflib) from SAM alignment files containing only reads aligning uniquely to the indicated reference. We expect to detect fewer polymorphisms than reported by QUAST because we are not calling insertions and deletions (Supplementary_Data_File). QUAST reports single-nucleotide polymorphisms (SNPs), as well as gaps in the assembly. A custom Python script, adjust_vcf_with_sam.py (Supplementary File 4), was created to ‘re-coordinate’ variant positions in the SAM alignment file of reads aligned against the de novo contigs to reflect their position in the HSV1 or HSV1 NR-references. In brief, the script uses the cigar string in the SAM file of the contigs aligned to the reference to realign the VCF file generated from the alignment of reads to the de novo assembly.

The genetic variant annotation and effect prediction toolbox (SnpEff) [34] was used to identify the distribution and potential effects of the identified variants on the HSV1 reference genome. These estimated effects are not biochemical predictions and should be interpreted with care. Biochemical certification is needed to make any conclusions about the functional impacts. Vcftools (https://vcftools.github.io/man_latest.html) was used to select genic regions from the VCF files.

### 2.8. Simulation Study

All possible simulated single end reads of 100, 250 and 500 bp were generated for the HSV1 and HSV1 NR-references where 80 nucleotide bases were randomly selected and modified to encode a different base, also determined at random. Simulated reads were aligned to their respective reference (BWA-MEM, single end) with the resulting SAM alignment file parsed to include only reads aligning uniquely. Simulated reads were assembled using the VelvetOptimiser Perl script with the resulting assemblies evaluated using QUAST, as was carried out for the non-simulated reads.

## 3. Results

### 3.1. Quality Control and Processing of High-Throughput Sequencing Reads

For this study, two HSV1 strains were sequenced. HSV1 strain 17syn+ is a clone of strain 17 and a well characterized laboratory strain, as well as the first HSV1 strain to be sequenced, first by Sanger sequencing (GenBank Accession Number: NC_001806.1 and [35]) and, more recently, by Illumina short read sequencing (GeneBank Accession Number: NC_001806.2). HSV 17∆CTRL2 is an HSV1 recombinant that was engineered (see Materials and Methods section and [19]) to contain a deletion of the CTRL2 CTCF binding motifs in the RL region [19]. Genomic sequences 17syn+ and 17∆CTRL2 were determined using viral DNA purified from cytoplasmic lysates of infected RSC followed by Illumina library construction and sequencing. Between 40 and 50 million 50 bp-paired end reads were obtained for both HSV1 strains (Table 1). Paired end read quality, evaluated using the FastQC tool [21], revealed that overall sequencing was successful (all data will be deposited into the National Center for Biotechnology (NCBI)’s Sequence Read Archive).

Both de novo assembly and variant discovery require that reads be of high quality and free of contamination. Reads derived from rabbit skin cells (used to propagate the virus) were identified and removed from the overall 17syn+ and 17∆CTRL2 reads by aligning paired end reads uniquely to the *O. cuniculus* genome (OryCun2.0, GenBank Accession Number: GCA_000003625.1) using Bowtie1 [24]. Unaligned reads and reads mapping ambiguously to the *O. cuniculus* genome (60.3% of total number of starting reads) were combined for subsequent analyses (Table 1). Retaining the rabbit ambiguous reads was to prevent premature removal of any HSV1-derived reads that may have aligned to repetitive regions of the rabbit host genome, in particular to rabbit CTCF regions. This is a particular problem with the herpesviruses because they contain several long clusters of CTCF binding motifs, which have similarity to their cellular orthologs [19]. Identical duplicate reads (≈1.3 million, 4.4% of the host-processed reads) were removed as these represent potential PCR artifacts or optical duplicates and would not contribute to de novo assembly, nor are they informative for variant identification. We refer to the remaining reads as host-processed reads (summarized in Table 1). There is an excess of paired end reads available for assembly and variant calling (>27 million for both isolates).

### 3.2. Non-Redundant HSV1 Genome

**Figure 2:**
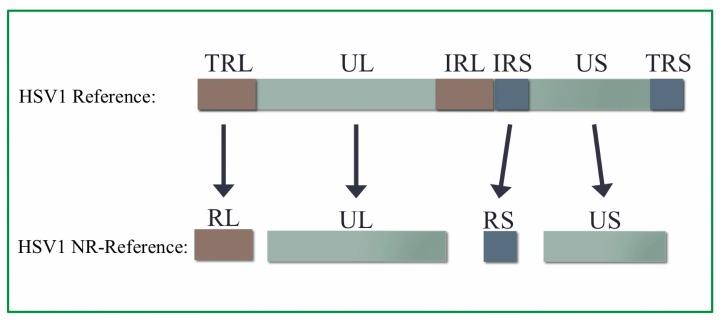
Full-length HSV1 reference genome and HSV1 NR-reference genome. The strain 17syn+ HSV1 genome (GenBank Accession Number: NC_001806) consists of two unique regions, referred to as unique long (UL) and unique short (US), and two repeat regions, referred to as repeat long (RL) and repeat short (RS). The repeat regions each have copies present at a terminal locus (TRL, TRS) and an internal locus (IRL, IRS) within the genome. The non-redundant NR-reference consists of the first copy of each of the repeat regions and both of the unique regions. Note: The figure is not to scale.

Approximately 21% of the HSV1 genome consists of a set of long inverted repeats (terminal repeat long (TRL) and internal repeat long (IRL)) and a set of short inverted repeats (terminal repeat short (TRS) and internal repeat short (IRS)) (Figure 2). Within each set of repeats, there are few sequence differences that distinguish terminal repeats from internal repeats. BLASTN alignments of the TRL and IRL regions reveals two variants, one of which is located in the CTa’m CTCF motif. Nucleotide Basic Local Alignment Search Tool (BLASTN) alignments of the TRS and IRS regions also identified variants, neither of which were associated with CTCF motifs. The high sequence identity across each set of repeats indicates that reads derived from these repeat regions are ambiguous with respect to which repeat they arise from and therefore cannot be mapped to a unique position. The high degree of sequence identity between the two copies of each repeat suggests recombination between these regions during HSV1 replication [36,37]. The existence of these repeats generates additional difficulties in genomic analyses, even by Southern blot, as HSV1 stocks consist of equal fractions of four different isomers of the genome, which differ in the relative position and orientation of the RS–US–RS segments, which can be on either the left or right side of the genome relative to the RL–UL–RL segments (Figure 2) [38].

To facilitate read alignment and interpretation, we generated a non-redundant version of the HSV1 genome (referred to as the HSV1 NR-reference) using the available strain 17syn+ genomic sequence (GenBank Accession Number: NC_001806). This HSV1 NR-reference consists of a single copy of the first repeat long (TRL, including the a’ sequence) and a single copy of the first repeat short (IRS) inverted repeat regions (Figure 2). Each region of the HSV1 NR-reference is contained in an individual FASTA record for downstream analyses. We note that the RL and RS regions were left fully intact as inter-strain variation within the CTCF motifs in these regions has been identified in alphaherpesviruses [39]. The length of the HSV1 NR-reference is 136,770 bp, 90% of the length of the full-length strain 17syn+ HSV1 reference.

### 3.3. De Novo Assembly of 17syn+

Given an HSV1 reference length of 152,261 bp (HSV1 NR-reference length of 136,770 bp) and a read length of 50 bp, redundancy (read number × read length/genome size) for 17syn+ was estimated to be 9464 and 10,536 for the HSV1 reference and HSV1 NR-reference, respectively. These values are well above the 8–10-fold minimum estimates for de novo assembly [40].

Velvet [17,25], a short-read assembler that uses de Bruijn graphs, was used to assemble the 17syn+ HSV1 genome from the host-processed reads. TheVelvetOptimiser Perl script [25] was used to automatically optimize the hash length, expected coverage and coverage cutoff parameters that are critical for successful Velvet assemblies (49, 278 and 0.26389, respectively). The final minimal overlapping length (hash length or k-mer) of 49 used in the Velvet assembly to join two reads was just under the read length of 50 bp. We hypothesize that the high coverage obtained in this experiment allowed for such a high k-mer; as k-mer length increases, sequencing depth must also increase since longer k-mers are less likely to overlap and more likely to contain sequencing errors [17]. The resulting Velvet contigs (157 total) were used as input into the CAP3 assembler [26], default settings), which uses an overlap algorithm (in contrast to de Bruijn graphs) to try and merge the Velvet contigs. Both the Velvet and Velvet-CAP3 assemblies were evaluated using QUAST [27] against the HSV1 reference to determine assembly metrics (Table 2). The N50 length (the length at which 50% of assembled nucleotides are found in contigs, 45,694 bp) and L50 count (the smallest number of contigs whose length sum produces N50, two contigs) were the same for both Velvet and Velvet-CAP3 assemblies. We note the presence of a single contig of 15,299 bp that QUAST identified as containing misassembly events relative to the HSV1 NR-reference for both Velvet and Velvet-CAP3 assemblies. QUAST classifies these misassemblies as events where the flanking sequence aligns to different ‘chromosomes’, in this case to different regions represented by separate FASTA records in the HSV1 NR-reference FASTA file. However, alignment of this contig to the HSV1 reference reveals that this contig aligns to a region of the HSV1 genome that overlaps the US and TRS regions and is therefore an artifact of evaluating the assembly using the NR-reference (recall that the HSV1 NR-reference is divided into four FASTA records with each record representing a different ‘region’ or ‘chromosome’). The additional merging of the Velvet assembly using CAP3 did not result in significant improvement of the assembly with respect to the QUAST assembly metrics despite the fewer number of total contigs in the Velvet-CAP3 assembly (Table 2).

The final Velvet-Cap3 assembly for 17syn+ contained 42 contigs greater than 500 bp, 15 of which aligned to both the full-length and non-redundant HSV1 references using BLAST-like alignment tool (BLAT) [28]. A BLASTN query of the 27 contigs not aligning to the HSV1 references against the NCBI nucleotide database/protein database (nr/nt) collection revealed sequence similarity to primarily *O. cuniculus* or predicted *O. cuniculus* sequences (15 of the 27; 56%). Assembly of host *O. cuniculus* contigs is not unexpected given that reads aligning ambiguously to the *O. cuniculus* host genome were retained for assembly. Other sequence subjects included *Schistosoma japonicum* (one of 27), *Acinetobacter baumannii* (one of 27), *Equus caballus* (one of 27) with the rest not aligning (33%). Importantly, none aligned to HSV1. The final assembly of the 17syn+ quality of the 15 contigs mapping to both HSV1 references was assessed using QUAST [27] and is consistent with the removal of *O. cuniculus* contigs (Table 2).

**Figure 3:**
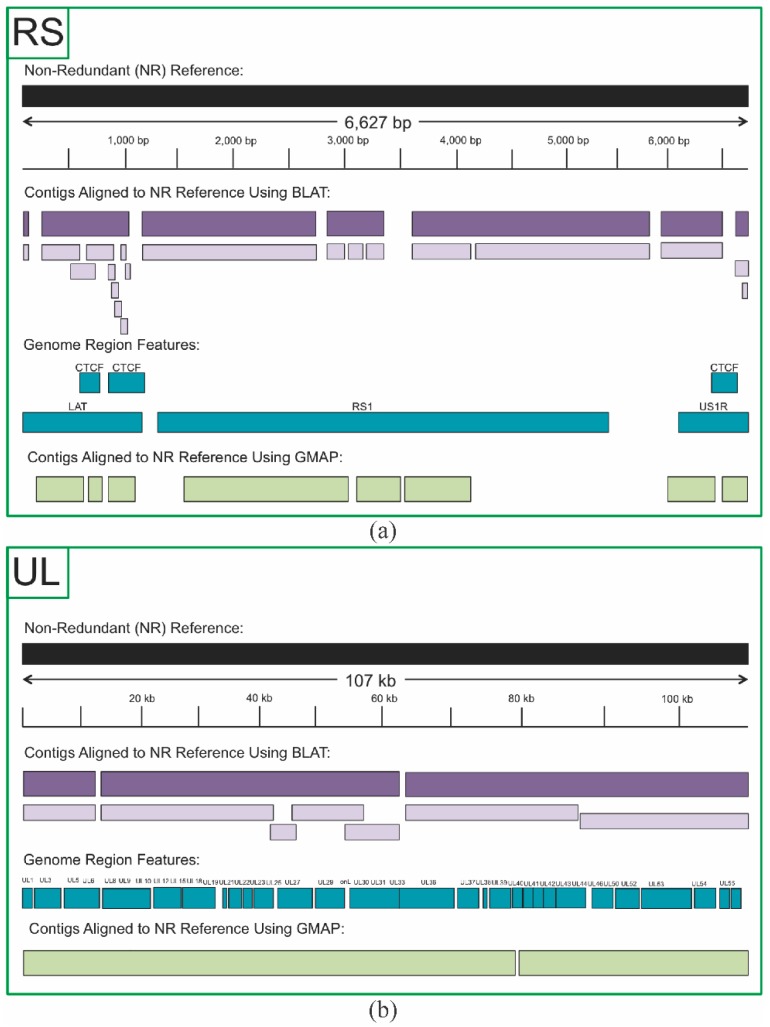
Contig alignment comparison using BLAST-like alignment tool (BLAT) and the Genomic Mapping and Alignment Program (GMAP) to the NR-reference. (**a**) Visualization of the alignment of 17syn+ contigs in the RS region of the NR-reference. Contigs aligned using BLAT are shown in light purple (consensus in dark purple) and contigs aligned using GMAP in green. Blue bars identify different genomics features; (**b**) Visualization of the alignment of 17syn+ contigs in the UL region of the NR-reference. Colors as in (**a**).

The final Velvet-CAP3 assembly was visually explored using IGV [32] against the HSV1 NR-reference. As expected, the RL and RS inverted terminal repeats were difficult to assemble de novo. Reiterated sequences within the inverted repeats can be visualized by comparing the output of BLAT and GMAP [30] in IGV. BLAT reports multiple alignments for individual contigs in addition to non-contiguous alignments, while GMAP outputs the best alignment for each full-length contig. Therefore, visualizing BLAT and GMAP output identifies regions of repetitive sequences (one contig will align to non-contiguous regions using BLAT). This can be seen in Figure 3 where discontinuous contig alignments are evident in the RS region (Figure 3a) as compared to the UL region (Figure 3b). We note that, not unexpectedly, the reiterated CTCF motif clusters in the repeat regions are difficult to assemble (Figure 3a).

**Figure 4:**
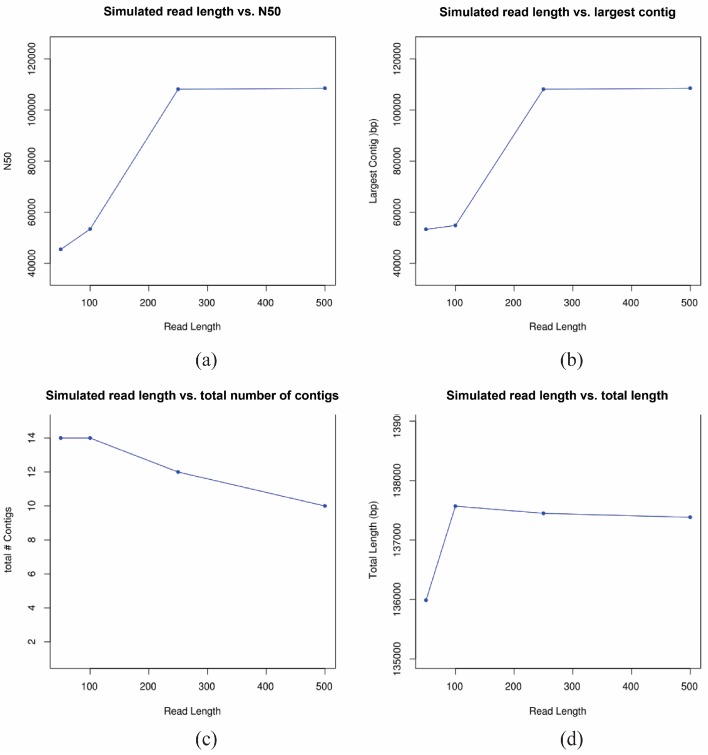
Effect of read length on de novo assembly metrics. De novo assemblies were generated using simulated reads of four different lengths (50, 100, 250 and 500 bp) and evaluated using QUAST. The effect of read length is shown for: (**a**) N50, (**b**) the largest contig, (**c**) the total number of contigs, and (**d**) the total length of the assembly. After 250 bp, the gain from longer read lengths levels off.

As longer read length is predicted to improve the assembly of repeat regions, we carried out a simulation study to determine the effect of increased read length on the de novo assembly of HSV1 and its repeat regions. Eighty positions in the HSV1 reference were randomly selected and the base changed to a random base prior to generating approximately 152,000 simulated single end reads for each read length of 50 bp, 100 bp, 250 bp and 500 bp. All possible reads for each read length were simulated and as such represent a ‘best case’ scenario, as any potential biases (e.g., GC content, homopolymers, etc.) are excluded from consideration. A lower bound of 50 bp was selected as this is the read length in the current study and 500 bp as the upper bound as this read length is slightly greater than the current maximum for standard Illumina library construction (MiSeq with maximum reads per run of 2 × 300 bp). Selected assembly metrics can be seen in Figure 4. There is an obvious effect of increasing the read length from 100 to 250 bp; N50 is the same length as the longest contig (Figure 4a), and the largest contig is almost double in length (Figure 4b). Therefore, there appears to be a ‘sweet spot’ with respect to read length for assembly of the HSV1 genome above which increasing the read length does not improve the quality of the de novo assembly. This is in agreement with simulations in other species that find that long reads may not improve transcriptome assemblies [41].

### 3.4. Alignments and Variant Calling

**Figure 5:**
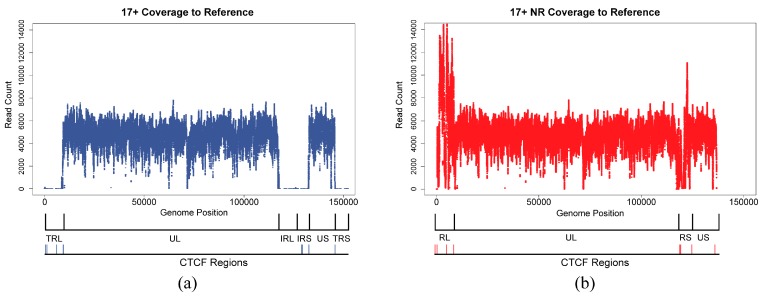
Coverage plots of reads aligning uniquely to the full-length HSV1 reference and the NR-reference. (**a**) The number of reads that aligned uniquely to all positions in the full-length HSV1 genome is on the *y*-axis (range from 0 to 10,307 with a median of 7548), while the genome position is on the *x*-axis. Genomic features are indicated under the plot including locations of CTCF motif clusters. Drops in alignment coverage for uniquely mapping reads are expected in the regions corresponding to the repeat regions. (**b**) The number of reads that aligned uniquely to all positions in the HSV1 NR-reference is on the *y*-axis (range from 0 to 17,324 with a median of 7597), and the genome is on the *x*-axis. Coverage is maintained in the representative repeat region. Genomic features are indicated as in (**a**).

Paired end host-processed 17syn+ and 17∆CTRL2 reads were aligned to the HSV1 reference genome or to the HSV1 NR-reference using BWA-MEM followed by the removal of reads aligning to multiple locations in the reference using an in-house Python script (Table 1). Coverage plots of read depth versus genome position for strain 17syn+ host-processed reads aligning uniquely to the HSV1 reference and the HSV1 NR-reference are shown in Figure 5. For 17syn+ reads aligning uniquely to the full-length HSV1 reference (Figure 5a), coverage is extremely low/non-existent in the repeat long and repeat short regions. We do not expect many reads to align unambiguously in this region, due to the nature of the repeats and the relatively short length of the reads (50 bp). In contrast, for 17syn+ reads aligned to the HSV1 NR-reference (Figure 5b), unique read alignments can be identified in the repeat regions. The reiterated sequences have higher coverage reflecting the increased copy number. Consistent with this, we find few 17syn+ reads and no 17∆CTRL2 reads aligning unique to the 135-bp core CTRL2 CTCF cluster (consisting of nine CTCF motifs) in the RL region of the HSV1 NR-reference where all reiterated sequences were kept. The HSV1 genome contains multiple tandem repeat clusters of CCCTC/CTCCC motifs, known binding motifs for the cellular insulator protein CTCF. Each tandem repeat contains the CCCTC/CTCCC pentanucleotide motif adjacent to an additional repetitive sequence unique to each cluster. These tandem repeat clusters correspond to the “reiteration sets” annotated in the 17syn+ reference genome (GenBank Accession Number: NC_001806.1) [35]. CTCF has been shown by chromatin immunoprecipitation to associate with several viral regions containing CTCF clusters during latent HSV-1 infection [19,42]. In addition, we note that more reads mapped uniquely to the HSV1 NR-reference as compared to the HSV1 reference (Table 1). These results are consistent with an overall reduction in ambiguity in the HSV1 NR-reference compared to the HSV1 reference. 

The SAM alignment files of host-processed 17syn+ and 17∆CTRL2 reads aligned uniquely to the HSV1 reference and to the HSV1 NR-reference were used to generate VCF files containing variant information, keeping only those variants with PHRED quality scores greater than 20 [22,23] using Freebayes [43]. The variants identified in the US and UL regions were identical irrespective of the reference used. Coverage in the RL and RS regions (HSV1 NR-reference) is much higher than coverage in the TRL/IRL and TRS/IRS regions (HSV1 reference) (Figure 5) as we map uniquely and there are fewer unique mapped reads in the HSV1 reference. Consequently, the number of variants identified when the HSV1 NR-reference was used was greater than when the full-length HSV1 reference was used (Table 3), irrespective of whether reads were generated from 17syn+ or 17∆CTRL2, and there are more variants in the RL and RS regions (HSV1 NR-reference) than in the TRL/IRL and TRS/IRS regions (HSV1 reference). Variants in 17∆CTRL2 are concordant with those in 17syn+, lending further evidence that these are no sequencing errors (Table 3).

Variants identified using the HSV1 reference were annotated [34] to identify any potential effects on HSV1 biology. Three of the 51 17syn+ variants identified using the HSV1 reference were removed by filtering on gene regions. The remaining 48 variants are predicted to result in 35 amino acid substitutions. Similarly, five of the 67 17∆CTRL2 variants identified using the HSV1 reference were removed by filtering on gene regions with the remaining 62 variants predicted to result in 38 amino acid substitutions.

All 80 simulated nucleotide variants were recovered when the full-length HSV1 was used, irrespective of read length (simulated reads length of 50–500 bp). When the HSV1 NR-reference was used, all variants were recovered except for one when a read length of 100 bp was evaluated. The one false negative was located 30 bp from the 3′-end of the RL region. This was at the edge of the simulation, and the number of simulated reads in this area is by definition low. Some of the reads did show the variants, but the algorithm did not detect the variant at this level of coverage, so this is an artifact of the way the simulation was designed.

**Figure 6:**
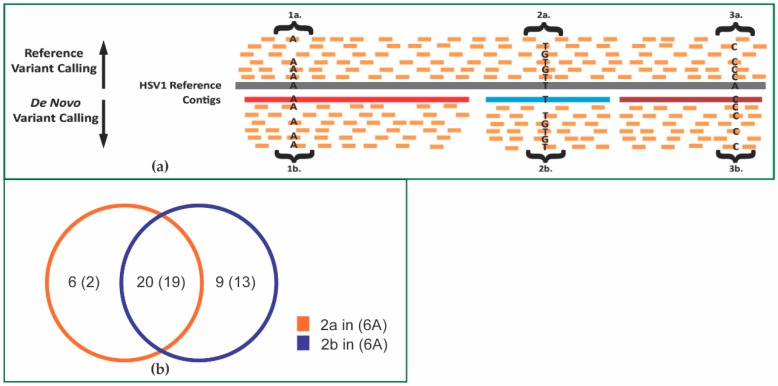
Determination of polymorphic sites. (**a**) Sites 1a, 2a and 3a represent specific loci with multiple reads aligned to the reference using a reference-based approach. (1a) A locus where all read sequences agree with the reference sequence. No variant is identified. (2a) A locus with polymorphic read sequences compared to the reference. A variant segregating in the population will be identified. (3a) A locus where all read sequences contain an alternate base compared to the reference. A variant divergent from the reference will be identified at this position. Sites 1b, 2b and 3b represent specific loci with multiple reads aligned to a contig reference generated from the reads. (1b) A locus where all read sequences agree with the reference contig sequence. No variant is identified. (2b) A contig locus with polymorphic read sequences compared to the contig consensus reference sequence. A variant will be identified. (3b) A contig locus where all read sequences contain the same base as the consensus reference sequence. No variant is identified. (**b**) Venn diagram of variants called with HSV1 NR-reference (or HSV1 reference) as in 2a above (orange) or 2b above (blue).

To determine if aligning to the de novo 17syn+ assembly improves variant discovery compared to aligning to the references, host-processed 17syn+ reads were aligned to the 15 final assembled Velvet-CAP3 contigs followed by the removal of ambiguous reads from the SAM alignment file and variant identification using Freebayes, again keeping only those variants with PHRED quality scores greater than 20. To compare variants identified using the contig sequences to those identified in the HSV1 references, a custom Python script was created that uses the cigar string in the SAM alignment file of the contigs aligned to the HSV1 reference or HSV1 NR-reference to ‘re-coordinate’ the VCF file containing the variant calls for reads aligned to the contigs (Supplementary File 4). The schematic in Figure 6a shows the variants identified, depending on whether a reference-based or de novo-based approach was used.

We identified 34 variants divergent from the HSV1 NR-reference (Table 4, Type 3a in Figure 6a). In the absence of this reference or when using a consensus reference, these loci would not be identified as variants solely from reads aligned to contig sequences (see 3b in Figure 6a). These variants, homozygous relative to a ‘stock’ genome reference, are of potential interest. Identifying and quantifying these ‘major’ variants may be advantageous, particularly in clinical settings. 

Across the RL, RS, UL and US regions, there were 26 segregating variants identified using the HSV1 NR-reference (Table 4, 2a as in Figure 6a) and 29 segregating variants identified solely using the contig sequences re-coordinated to the HSV1 NR-reference (Table 4, 2b as in Figure 6a). Twenty of these variants were shared across both sets (Figure 6b) with 19 of the 20 located in the UL region. 

We also note the presence of variants unique to the HSV1 NR-reference aligned reads (2a in Figure 6b) and unique to the contig aligned reads (2b in Figure 6b). Of the six variants unique to the HSV1 NR-reference, four were located in the RL region and one in the RS region. Of interest, two of these six variants were in CTCF motifs. For five of these six variants, there was no contig mapped, making it impossible to call variants in the contig reference. These variants likely represent false negatives due to incomplete coverage of the genome in the de novo assembly and represent the risk associated with using a de novo assembly technique. 

The inconsistency between the variants identified using the HSV1 NR-reference and the contigs can be explained by the presence of false negative variants in the reference-based approach or by the presence of false positive variants in the de novo assembly approach. Of the nine variants unique to the contig reference, most (six of the nine) were located in the UL region, one in the RS region and two in the RL region, one of which was located adjacent to a CTCF motif. All nine variants are in regions of high GC content. Four of the nine have no BLAST hit for the 50 bp sequence (25 bp up and downstream of the variant as query sequence) to the HSV1 NR-reference, indicating that these variants are false positives in the de novo assembly. One variant is in a 50 bp window that is found in at least two other locations in the HSV1 NR-reference. The remaining three variants are adjacent to a small 1 bp gap in the alignment of the contig to the HSV1 NR-reference. The false identification of variants when gaps are nearby has been documented [44]. We conclude that the variants unique to the de novo assembly are likely false positives.

## 4. Discussion

High-throughput sequencing has afforded the ability to rapidly and inexpensively sequence large viral genomes, such as the herpesviruses. There has been an increasing interest in using this technology to sequence clinical isolates, as well as to conform the genetic integrity of recombinant virus constructs. However, the assembly of complete herpesviral genomes from small HTS reads is not without challenges. Chief among the difficulties are: (1) the existence of repeat regions of the genome; (2) numerous reiterated sequences, such as the CTCF motif clusters; and (3) the fact that there is heterogeneity in viral populations both in cultured virus, as well as in human clinical isolates.

In this study, we sought to compare two common methods of assembling the DNA sequence, de novo and reference-based assembly. We applied these two methods to a common laboratory problem: comparing a viral recombinant with its wild-type parent to determine sequence differences. By choosing a parent viral strain that had been extensively sequenced, it allowed us to sensitively compare these two sequence assembly approaches for their abilities to detect differences. From these studies, we made several important observations.

De novo assembly algorithms are complicated by the presence of reads representing host DNA. An initial alignment to host DNA reduces the complexity of the genome to be assembled and avoids misassembly of repeat regions that are similar between the species. De novo assembly has the advantage that direct sequencing from a clinical sample could allow identification of a pathogen without prior knowledge of the viral infection, potentially leading to more rapid diagnostic tools. However, reiterated regions present challenges in de novo assembly, even when coverage levels are high, with an excess of variants falsely identified. The false positives in variant calling from the de novo assembly are largely due to mapping errors resulting from aligning to the consensus sequence rather than a complete genome. The reference quality is known to be critical along with stringent mapping, particularly with poorer, fragmented references [45]. While this paper focuses on high throughput short read sequences, there is new long read technology and observed improvements in reference quality due to combined assemblies from long and short reads [46]. Although, even with long read technologies or indeed Sanger sequencing, repeats and reiterated sequences are problematic, and assembling them correctly may take more resources than most sequencing projects have allocated. Further, it is arguable whether this is a good use of reagent, when a high quality complete genome sequence is available.

Reference-based approaches benefit from the reduction of complexity in creating a non-redundant reference genome where repetitive regions are removed from all but a single place in the genome. Repetitive regions can then be quantitated by read mapping. If desired, copy number variation approaches could be used to identify polymorphisms in repeat number (reviewed in [47]). Using this strategy, more total reads map unambiguously to the genome. Variants identified in the repeat regions likely reflect variation among repeats in the genome. These variants may have arisen during recombination between the inverted repeats [36,38] or by other mechanisms. Recombination and segment inversion of HSV1 genomes for the formation of genome isomers have been done [48,49]. Unfortunately, genome isomers cannot be quantified when a reference-based approach is implemented.

In a clinical setting, the ability to sequence virus directly without the need for culture may lead to the ability to estimate the polymorphism rates in natural populations of virus. In these cases, de novo assembly has a distinct advantage in that the virus (or other pathogen) does not need to be identified in order to proceed, potentially providing a faster method of diagnostics. However, the low titer of many viruses still requires an enrichment phase prior to HTS to ensure enough viral reads for assessment. For now, the viral identification step still precedes sequencing.

Ever since the first HSV-1 genome sequence was completed [35,50], difficulties in accurately assembling error-free and unambiguous genome sequences have been apparent. This was highlighted by sequencing errors in a high GC-rich region of the RL segment that led to the initial report that the important neurovirulence gene *ICP34.5* was present in some strains of HSV-1, but not strain 17, when it was later demonstrated that *ICP34.5* was present in strain 17 [51]. Therefore, even with the relatively long read length and high quality afforded by Sanger sequencing, the complexity of the herpesvirus genomes presents a sequencing challenge. The smaller read lengths common in Illumina sequencing protocols have difficulty resolving reiterations that are common in the Herpesvirus genomes, especially the CTCF clusters. While our simulations show that longer read lengths are better able to facilitate reliable assembly, they do not completely resolve these problems. Also, difficult in de novo assembly is the fact that large segments of the genomes are present as two copies, which may or may not be identical. The inability to resolve these problematic regions easily, even with Sanger sequencing, increases the time spent in assembling and validating the sequences of HSV genomes. While de novo assembly has been employed with accuracy and success [52], the results of our comparisons here suggest that a significant savings in time and efficiency can be gained by a reference genome-based assembly. A de novo approach may be preferable with more distantly-related isolates or clinical isolates that may contain recombination between two viruses, such as HSV-1 and HSV-2. However, for routine sequencing of different strains or isolates of HSV-1, or characterization of segregating alleles in a population of HSV-1 or for characterizing laboratory generated recombinants and mutants, a reference-based assembly will quickly generate accurate results, even with short (50 bp) reads. Those wishing for an exhaustive survey may still choose to pursue both avenues and may consider manual intervention to improve assembly [5].

## Figures and Tables

**Table 1:** Illumina sequencing and processing. The HSV1 reference (GenBank Accession Number: NC_001806) was used intact and then reduced to only non-redundant (NR) where each repetitive region was included only once in the reference. The coordinates for the NR-reference are in Supplementary File 5.

Reads	17syn+	17∆CTRL2
total # paired end reads	49,967,108	43,635,207
# paired end aligning (unique + ambig) to Oc (%)	23,418,917 (46.9%)	254,356 (6.9%)
# paired end non-host reads (unaln + ambig)	30,149,765 (60.3%)	3,414,197 (94.0%)
# paired end host-processed reads	28,822,236	34,263,711
# reads aligning uniquely to HSV1 reference	18,802,764	49,774,582
# reads aligning uniquely to HSV1 NR-reference	21,418,760	55,660,042

Ambig: Ambigious reads, Unaln: Unaligned reads.

**Table 2:** 17syn+ De novo assembly metrics using the HSV1 reference (GenBank Accession Number: NC_001806). Assembly metrics generated by QUAST [27] are based on contigs greater than or equal to 500 bp in length, unless otherwise noted.

Metric	Velvet Contigs	Velvet-CAP3 Contigs	HSV1_Mapped_Contigs
Total # of Contigs	157	105	15
# Contigs ≥ 1000 bp	27	27	10
Largest Contig (bp)	53,719	53,719	53,719
Total Length ≥ 0 bp	188,444	183,313	135,829
Total Length ≥ 1000 bp	162,124	162,124	131,913
N50	45,694	45,694	45,694
L50	2	2	2
Genome Fraction (%)	97.37	97.44	97.44
Duplication Ratio	1.018	1.018	1.018
% GC	64.34	64.32	67.41
NR-Reference Length (bp)	136,770	136,770	136,770
Reference % GC	67.56	67.56	67.56
# N’s	8303	8303	1907
# Mismatches + Indels	82	83	83

N50: Length at which 50% of assembled nucleotides are found in contigs; L50: Smallest number of contigs whose length sum produces N50.

**Table 3:** The number of variants depends on reference construction. Using a non-redundant reference (HSV1 NR) increases the number of variants identified.

Reads	Reference	# Variants in RL	# Variants in RS	# Variants in US	# Variants in UL	Total # Variants
17syn+	HSV1	2	1	2	46	51
17syn+	HSV1 NR	9	3	2	46	60
17∆CTRL2	HSV1	2	1	2	43	48
17∆CTRL2	HSV1 NR	8	3	2	43	56

RL: Repeat long; RS: Repeat short; US: Unique short; UL: Unique long.

**Table 4:** Reference variant calling vs. de novo variant calling. Number of variants identified using the indicated alignment strategy (as indicated in Figure 6) for variants called against the HSV1 NR-ref or the HSV1 reference (in parentheses).

Alignment Strategy	# Variants in RL	# Variants in RS	# Variants in UL	# Variants in US	# Variants Total
2a: segregating variants	5 (0)	1 (1)	19 (19)	1 (1)	26 (21)
2b: segregating variants	3 (4)	1 (3)	24 (24)	1 (1)	29 (32)
3a: divergent variants	4 (2)	2 (0)	27 (27)	1 (1)	34 (30)

## Supplementary Materials

The following are available online at www.mdpi.com/1999-4915/9/8/226/s1, Supplementary_File_1.txt (FASTA file of HSV1 NR-reference), Supplementary_File_2.txt (FastqSplitDups.py Python script), Supplementary_File_3.txt (BWASplitSam.py Python script), Supplementary_File_4.txt (Adjust_vcf_with_sam.py Python script), Supplementary_File_5 (BED file for HSV1 NR-reference) and Supplementary_Data_File (4 VCF files containing single nucleotide variants).
