# Supplementary material for: Reliable Detection of Herpes Simplex Virus Sequence Variation by High-Throughput Resequencing
Source: Viruses. 2017 Aug 16;9(8):226. doi: 10.3390/v9080226 (PMC5580483; doi:10.3390/v9080226)
Supplement: Supplementary file 1 [file viruses-09-00226-s001.tgz › Supplementary_Data_File/ReadMe_contents_of_Supplementary_Data_File.docx]

Supplementary Data File contents:

(1) VCF file of 17syn+ read alignment against HSV1 reference:

SNPS_only_17p_freebayes_clean_distinct_2_notNR.recode.vcf

(2) VCF file of 17syn+ read alignment against HSV1 NR-reference:

SNPS_only_17p_freebayes_clean_distinct_2_NR.recode.vcf

(3) VCF file of 17∆CTRL2 read alignment against HSV1 reference:

SNPS_only_deltaCTRL2_freebayes_clean_distinct_2_notNR.recode.vcf

(4) VCF file of 17∆CTRL2 read alignment against HSV1 NR-reference:

SNPS_only_deltaCTRL2_freebayes_clean_distinct_2_NR.recode.vcf
